# Auto-Rad: End-to-End Report Generation from Lumber Spine MRI Using Vision–Language Model

**DOI:** 10.3390/jcm13237092

**Published:** 2024-11-23

**Authors:** Mohammed Yeasin, Kazi Ashraf Moinuddin, Felix Havugimana, Lijia Wang, Paul Park

**Affiliations:** 1 Department of EECE, The University of Memphis, Memphis, TN 38152, USAlwang3@memphis.edu (L.W.); 2 Department of Neurosurgery, College of Medicine, The University of Tennessee Health Sciences, Memphis, TN 38163, USA; ppark4@uthsc.edu

**Keywords:** lumber spine MRI, lumbar spinal stenosis, vision–language model, Generative Image-to-Text, automated radiology report generation

## Abstract

**Background:** Lumbar spinal stenosis (LSS) is a major cause of chronic lower back and leg pain, and is traditionally diagnosed through labor-intensive analysis of magnetic resonance imaging (MRI) scans by radiologists. This study aims to streamline the diagnostic process by developing an automated radiology report generation (ARRG) system using a vision–language (VL) model. **Methods:** We utilized a Generative Image-to-Text (GIT) model, originally designed for visual question answering (VQA) and image captioning. The model was fine-tuned to generate diagnostic reports directly from lumbar spine MRI scans using a modest set of annotated data. Additionally, GPT-4 was used to convert semistructured text into coherent paragraphs for better comprehension by the GIT model. **Results:** The model effectively generated semantically accurate and grammatically coherent reports. The performance was evaluated using METEOR (0.37), BERTScore (0.886), and ROUGE-L (0.3), indicating its potential to produce clinically relevant content. **Conclusions:** This study highlights the feasibility of using vision–language models to automate report generation from medical imaging, potentially reducing the diagnostic workload for radiologists.

## 1. Introduction

Chronic lower back and leg pain, frequently resulting from a condition known as lumbar spinal stenosis (LSS), is a widespread affliction that affects millions of people worldwide. The diagnosis of LSS relies on a comprehensive evaluation of the patient, including symptoms and clinical examinations, magnetic resonance imaging (MRI) is instrumental in identifying physical spinal abnormalities. Radiologists scrutinize MRI scans in both axial and sagittal views to spot signs of spinal canal narrowing or structural anomalies like disc protrusion and bulging, often leading to nerve root compression and related pain symptoms [1,2,3,4].

The comprehensive analysis of MRI scans by experts in radiology is inherently laborious and time-intensive, resulting in treatment delays and increased healthcare costs. In response to these issues, we built an end-to-end system designed to assist radiologists in assessing LSS from MRI scans and automatically generate relevant reports. Our approach involved the utilization of advanced vision–language (VL) deep learning models to generate comprehensive diagnostic reports from lumbar spine MRI scans. These models, with their ability to learn from a wide array of images, have the potential to significantly lighten the workload of medical professionals, speed up the diagnostic process, allow faster initiation of treatment plans, and mitigate associated costs.

The application of deep learning (DL) models in medical imaging and reporting has seen considerable progress in recent years [5]. These models have shown promising results in generating evaluation reports from various imaging techniques, such as X-rays [6], computerized tomography (CT) [7], and MRI [8]. Specifically, Han et al. demonstrated a weakly supervised framework for lumbar spine evaluation that uses object-level annotation to generate evaluation reports [8]. In this paper, we show that VL models, pre-trained in image captioning settings, can be fine-tuned to generate coherent, easily readable reports in an end-to-end fashion.

Although end-to-end deep learning models exhibit impressive performance in various applications, they typically require abundant annotated samples for training. In the medical imaging field, the acquisition of large amounts of annotated data is often impractical due to privacy concerns, expense, and the need for expert input. The advancement of self-supervised learning (SSL) techniques has mitigated this issue, enabling the development of robust general vision models that can be fine-tuned for specific downstream tasks using fewer samples. Studies such as the one conducted by Azizi et al. demonstrated that SSL models initially trained on natural images, such as those from the ImageNet dataset, can perform impressively well when fine-tuned with medical images for specific tasks [9]. This underscores the value of using SSL approaches in domains where annotated data may be scarce or expensive to obtain, such as medical imaging.

We used the Generative Image-to-Text (GIT) [10] model to build an end-to-end system named Auto-Rad for LSS diagnostic and reporting. GIT was trained using millions of image–caption pairs, with conditioning on both the image and textual input. GIT utilizes a transformer decoder conditioned on image and text embeddings. GIT image encoder is trained in a contrastive VL SSL setting [10], which produces embeddings that contain textual and image contexts. The embeddings are used to subsequently train a transformer decoder from the ground up to generate captions. GIT has achieved top-tier performance in various tasks such as visual question answering (VQA) and image captioning. In our study, we demonstrate that this model can be repurposed to function as an automatic report generator based on lumbar spine MRIs with a considerably small sample size (approximately 1500), thus showcasing the possibilities for end-to-end systems even when annotated data are scarce.

We used natural language generation (NLG) and image captioning metrics to evaluate the Auto-Rad models’ capacity to generate coherent, semantically accurate, and grammatically well-structured reports. We further conducted empirical and topic-level evaluation to asses the model performance for LSS diagnosis. Despite the room for improvement in LSS assessment performance, promising results present a compelling case for further research. In the following sections, we elaborate on the steps of our experiment, present our results, and discuss our model evaluation, its limitations, and potential future directions.

## 2. Related Works

DL models have been effectively applied to detect and classify lumbar spinal stenosis, achieving high accuracy and interobserver agreement comparable to that of radiologists [11,12]. CNNs have shown high diagnostic accuracy in identifying severe central lumbar spinal stenosis [13], automatic segmentation of spine MRI scans [14,15], and vertebrae detection [16]. Various DL approaches have also been employed for automatic radiology report generation. These include tailored CNNs and MobileNets for volume-level and question-specific features [7], encoder–decoder models with contrastive learning [17], and contextual embeddings like DistilBERT combined with hierarchical LSTM [18]. Other approaches encompass BERT-based architectures with multimodal attention [19], multimodal recurrent models with attention mechanisms [20], and encoder–decoder frameworks with novel text modeling and visual feature extraction [21]. Additionally, some models focus on multi-view image fusion and medical concept enrichment [22] or utilize memory-augmented sparse attention and Medical Concept Generation Networks [23]. These methods have demonstrated significant performance improvements, such as high BLEU scores and classification accuracy.

Most existing medical report generation models are not trained in an end-to-end manner; instead, they are pre-trained for tasks like detection, classification, or segmentation to provide the model with more context and reduce diagnostic errors. While this approach can be effective, it often requires a large amount of annotated data for pre-training and downstream report generation. We demonstrate that in situations where such an abundance of samples is unavailable, VL models like GIT [10], pre-trained on natural image captioning, can still generate coherent and diagnostically accurate reports.

## 3. Methodology

### 3.1. Dataset

The data employed for our experiment were obtained from a collection curated by Sud et al. [24]. This dataset includes clinical MRI scans and corresponding radiologist reports from 515 anonymous patients. For each individual, images of the sagittal and axial views of the lower three vertebrae and intervertebral discs (IVDs) were captured. These views incorporated both T1- and T2-weighted scans.

**Figure 1:**
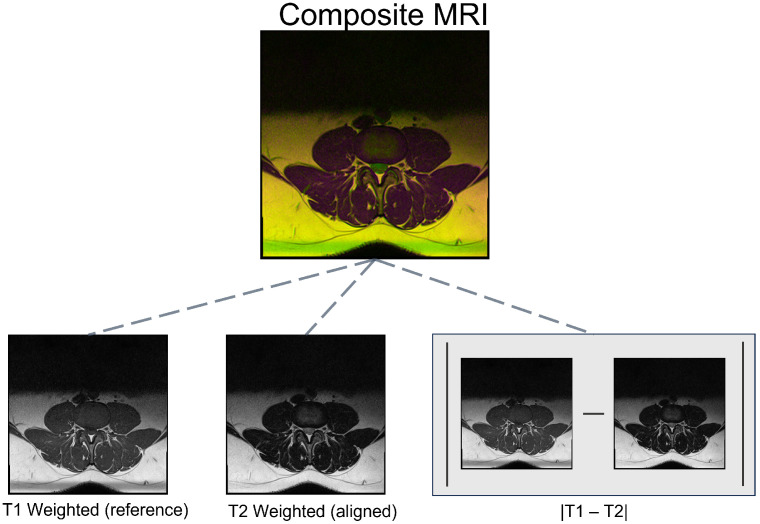
Breakdown of the composite MRI images. The composite MRI image consists of three channels: the T1-weighted MRI (red), the registered T2-weighted MRI (green), and the Manhattan distance between the T1 and T2 images (blue).

Al-Kafri et al. used T1- and T2-weighted axial MRI scans of this dataset to create a composite representation by aligning the images and stacking them together [25]. This composite image, composed of three channels, incorporated T1-weighted images, aligned T2-weighted images, and the Manhattan distance between the two as the first, second, and third channels, respectively [25] (see Figure 1). The composition process resulted in 1545 RGB MRI images, where each patient is associated with three composite MRI images. To fine-tune the GIT model, we used these composite images in conjunction with their corresponding clinical reports.

### 3.2. Text Transformation Using GPT-4

**Figure 2:**
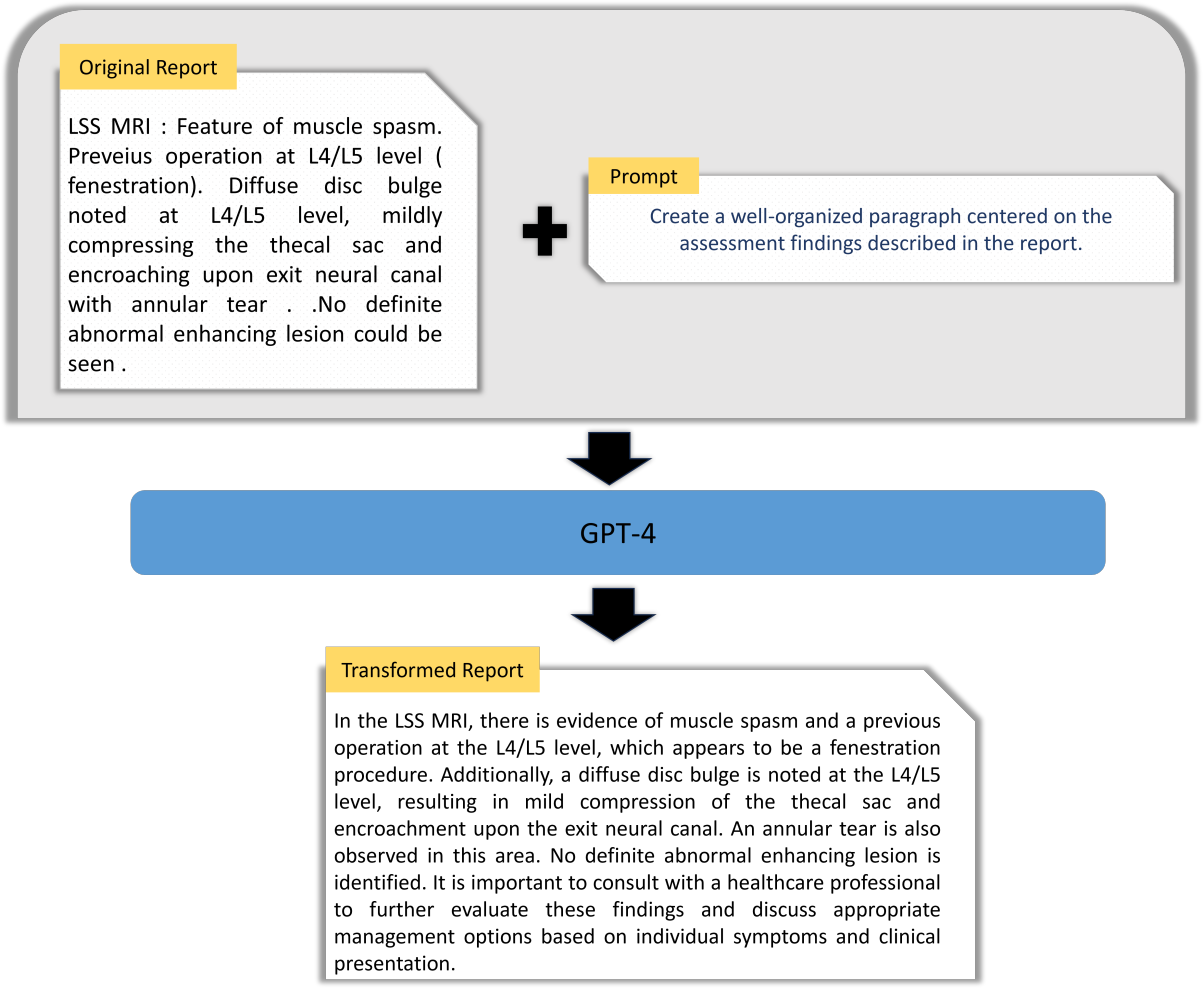
Process of employing GPT-4 for text transformation of original radiologists’ reports, generating a singular paragraph that encapsulates all the assessments found within the reports.

The radiologists’ reports we utilized comprised semistructured clinical annotations from expert analysis of MRI scans. These reports often contained spelling and grammatical errors, which presented a challenge for effective training of the GIT model, particularly since this model was originally trained using structured human annotations. To address this, we harnessed the power of GPT-4 [26] to transform these semistructured clinical notes into more coherent paragraph structures, which the GIT model could more readily ingest. Each of the 515 reports was used as a prompt for GPT-4, with additional instructions to reformulate the assessment in the report into structured paragraphs. Figure 2 presents an overview of the report transformation process utilizing GPT-4. The subsequent section delves into the prompt engineering methodology employed to facilitate this transformation.

#### Prompt Engineering Approach

- **Initial prompt structure**: The initial prompt presented to GPT-4 for each report contained the full clinical note as input, followed by an instruction to “rephrase into a coherent, single-paragraph summary focusing on the assessment”.
***Prompt example:***

“L4-L5: diffuse disc bulge noted, compressing the thecal sac and exit canals. Convert this to a coherent paragraph summarizing the key assessment findings”.

- **Challenges in text transformation**:
  − ***Inconsistent terminology***: Radiologists often used varying terms to describe similar findings (e.g., “No significant thecal sac compression” vs. “adequate thecal sac”). This inconsistency required refinement in the prompts to encourage GPT-4 to use standardized language.
  − ***Abbreviations and shorthand***: Reports contained medical shorthand (e.g., “thecal sac” as “TS”), which was not always uniformly understood by GPT-4. To address this, prompts were modified to ask GPT-4 to expand medical abbreviations where possible.
  − ***Spelling and grammatical errors***: The model’s performance was occasionally hindered by spelling errors in the raw data, necessitating additional preprocessing instructions to GPT-4 to ensure it corrected these issues while maintaining the original meaning.
- **Prompt development and standardization**: We refined the prompts iteratively in order to reach a standardized template for this task. Outputs were evaluated for coherence and clinical accuracy, with adjustments made to ensure inclusion of critical details like muscle spasms, disc herniation, bulging, thecal sac compression, and Ligamentum flavum hypertrophy. The final standardized prompt directed GPT-4 to summarize and rephrase reports, correct spelling/grammar, expand abbreviations, and maintain consistent terminology.
  − ***Final standard template for prompt***: “Given the radiologist’s clinical assessment report below, rephrase and transform the information into a structured, coherent paragraph that corrects any spelling or grammatical errors, expands abbreviations, and maintains clinical accuracy. Ensure the paragraph clearly communicates key findings related to disc conditions, nerve root compressions, muscle spasms, and any other relevant observations. Please use consistent terminology, avoid omitting any clinical details, and format the output as a concise paragraph”.

### 3.3. Stratified Topic-Based Data Splitting

**Figure 3:**
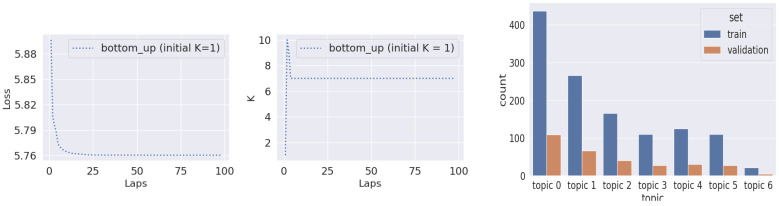
(**Left**): Diagnostics of the HDP training. The left figure shows the progression of variational loss across 100 laps. The right figure demonstrates the number of topics generated in each lap. The model is initialized with K = 1 latent topic and, after 100 laps, converges to 7 topics, which we used for stratifying our dataset. (**Right**): Topic-wise distribution of samples in the training and validation sets. The data are split to maintain the proportionate amount of samples in each topic for the train and validation sets.

Our task, framed as an image description generation problem, posed a challenge in ensuring that all variations of lumbar spine assessments were adequately represented in the training and validation datasets. To overcome this, we employed the hierarchical Dirichlet process (HDP) [27] to identify latent topics within the 515 radiologist reports. The model was trained from the bottom up with an initial topic number of K = 1, using a bag-of-words representation along with a multinomial prior. To improve computational efficiency, we utilized birth and merge moves within the memoized variational inference algorithm [28,29]. Upon convergence, the model identified seven latent topics, as illustrated in Figure 3.

The latent topics, once identified, were associated with each radiologist’s report. This association was based on the maximal posterior probability of topics given the words in each report, thereby effectively categorizing the type of assessment conveyed in the reports. We then partitioned the dataset into training and validation sets in an 80–20 split, using the identified latent topics for stratification. Figure 3 shows the distribution of sample sizes in the training and validation sets.

### 3.4. Report Generation Model

**Figure 4:**
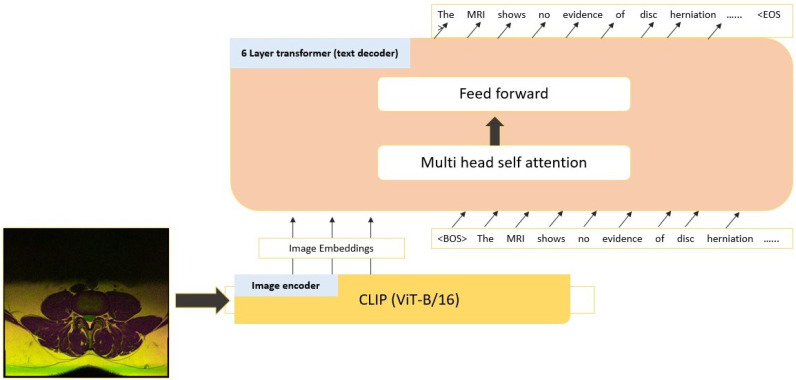
Architecture of the ‘GIT-base’ model. Comprises a CLIP model that utilizes a vision transformer (ViT-B/16) [10,31], and a 6-layer transformer decoder for text generation. The CLIP module generates 768-dimensional image embeddings which, together with the text from the report, serve as the input for the text decoder. During fine-tuning, the parameters of both the image encoder and the text decoder are adjusted.

The base model for Auto-Rad is accessible on Hugging Face [30] as ‘microsoft/git-base’. The model is similar to the ${\mathrm{GIT}}_{B}$ model from Wang et al. [10]. It uses an image encoder, which is a version of the CLIP model [31] with a ViT-B/16 vision transformer, and a text decoder with six layers and multi-head self-attention [32]. The decoder combines image embeddings from CLIP with original radiologist notes as inputs to generate assessment reports. It was initially trained in a ‘teacher forcing’ manner [33] using 10M image–text pairs for image captioning [10]. Figure 4 provides a simplified diagram of the model architecture and the fine-tuning process.

**Figure 5:**
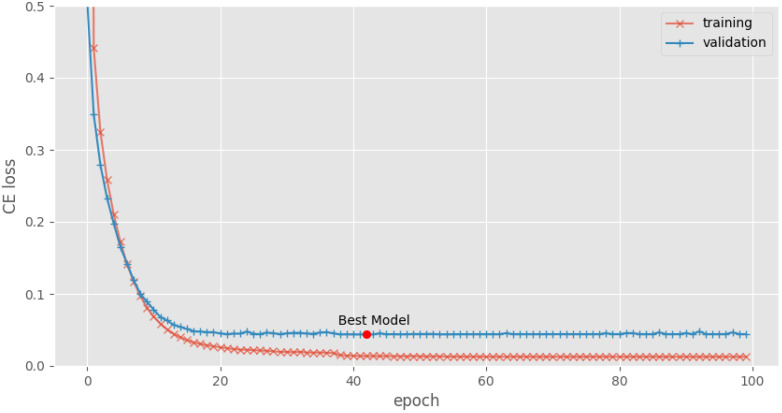
Progression of training versus validation loss during the model’s training phase.

Fine-tuning the model involved a dataset of 1236 MRI–report pairs, while 309 additional pairs were set aside for validation. Utilizing a NVIDIA RTX A4500 GPU, the training process spanned over 100 epochs with a batch size of 8 and an initial learning rate of $5\;\times \;10^{-5}$. We used cross-entropy (CE) loss and the AdamW [34] optimizer during training, with plateau learning rate reduction enabled. The model checkpoints were saved whenever a decrease in the validation error was observed. The progression of the training and validation cross-entropy (CE) losses across the epochs is illustrated in Figure 5. The model checkpoint shows that the model reached its minimum validation loss at the 42nd epoch (marked by the red circle). For subsequent evaluations, we selected the model from this specific checkpoint.

### 3.5. Evaluation Process

We primarily employed natural language generation (NLG) and image captioning metrics to evaluate the model. We employed a variety of measures, including ROUGE [35], BLEU [36], METEOR [37], BERTScore [38], perplexity [39], CIDEr [40], and SPICE [41]. We further empirically tested the quality of the generated reports in addition to NLG and image captioning metrics. We focused on 3 assessment criterion for the empirical evaluation:

1. **Diagnostic completeness (DC)**: Defined as the fraction of original diagnoses reflected in the report produced by the model, it essentially determines the presence of original diagnostic content from the radiologist’s report in the output generated by the model.
2. **Novel diagnostic detection (NDD)**: Measures the proportion of new diagnoses found in the report generated by the model but absent in the original radiologist’s report. Assesses whether the model has added any diagnostic content not initially found in the original report.
3. **Diagnostic correspondence (DCorr)**: Represents the percentage of discrepancies in the diagnoses between the model-generated and the original reports. Assesses the degree of agreement between the diagnoses in the reports generated by the model and the original diagnoses from the radiologist’s report.

If $D_{o}\;\in \;W_{o}$ and $D_{g}\;\in \;W_{g}$ represent the set of diagnoses in the original and generated report, respectively, where $W_{o}$ and $W_{g}$ embody the sets of words describing the prognosis in the original and generated reports, respectively, then DC, NDD and DCorr are formulated as

(1) $$\begin{array}{cc} DC & =\frac{|D_{o}\cap D_{g}|}{|D_{o}|}. \end{array}$$

(2) $$\begin{array}{cc} NDD & =\frac{|D_{g}-D_{o}|}{|D_{g}|}. \end{array}$$

(3) $$\begin{array}{cc} DCorr & =\frac{|D_{o}\cap D_{g}|}{|D_{g}|}. \end{array}$$

We further used the HDP topic model purposed for data stratification to compare the distribution over topics between the original and generated reports. The topic-level comparison is used to quantify the consistency in theme and content between the original radiologist reports and the model-generated reports, ensuring that the model effectively captures and replicates the key informational aspects from the original data in its generated output. Beyond assessing the grammatical correctness or readability of the generated reports, this approach provides an additional layer of validation for the model’s performance.

To compare the topic-level distributions, we first acquired posterior distribution of topics for the each of the original and generated report pairs. We then used Jensen–Shannon divergence (JSD) [42] and earth mover distance (EMD) [43] to compute the similarity in latent topic distribution between the original and generated texts. If $\theta_{o}$ and $\theta_{g}$ represents the latent topic distribution of the original and generated reports, then

(4) $$JSD\left(\theta_{o}∥\theta_{g}\right)=\frac{1}{2}\left(KL\left(\theta_{o}∥M\right)+KL\left(\theta_{g}∥M\right)\right).$$

(5) $$EMD\left(\theta_{o},\theta_{g}\right)=\min_{\gamma }\sum_{i,j}\gamma \left(i,j\right)\cdot d\left(i,j\right).$$

For Equation (4), *M* is the average distribution between $\theta_{o}$ and $\theta_{g}$ and KL denotes the Kullback–Leibler divergence [44]. For EMD (Equation (5)), γ is the transportation plan that specifies how much mass from each topic in $\theta_{o}$ is transported to each topic in $\theta_{g}$ and d(i,j) is the distance (cost) between topics *i* and *j* in terms of their probability distributions.

## 4. Results

### 4.1. Model Evaluation

In the following section, we outline the in-depth performance of the Auto-Rad model utilizing well-established NLG and image captioning metrics. The model’s performance, as assessed by these metrics, is consolidated in Table 1.

Auto-Rad demonstrates satisfactory performance in recognizing semantic patterns in the generated reports, as evidenced by its METEOR score of 0.37 and BERTScore of 0.886. Despite this, the model has difficulties mirroring sentence-level phrasing, particularly bigrams, reflected in the low ROUGE-2 score of 0.185. The CIDEr and BLEU scores further support the notion that the model’s generated reports are not in perfect alignment with the original report at a phrasal level.

However, the good METEOR score reveals that despite different phrasings, the generated captions retain a certain level of semantic consistency with the reference captions. Additionally, with an SPICE score assessment based on scene graphs, we can infer that the model successfully captures roughly 29% of the scene graph tuples in the reference captions, signifying a certain level of semantic understanding.

The model’s low perplexity score of 1.045 implies minimal uncertainty in report generation, but might be a consequence of having a limited sample size with small variation.

### 4.2. Empirical Evaluation

We conducted an empirical evaluation on the 100 randomly selected samples from the test dataset. During the evaluation, we computed three key diagnostic metrics, DC, NDD, and DCorr, for each pair of original and generated reports. The mean values of these metrics were calculated to provide an overall summary of the model’s ability to generate accurate diagnostic content in its reports. The results indicated that the model achieved a mean DC of 24.7%, suggesting that it captured approximately 24.7% of the original diagnostic information present in the generated reports. Furthermore, the NDD metric yielded a mean value of 69.8%, indicating that the model introduced new diagnostic content, not present in the original reports, in around 69.8% of the generated reports. Lastly, the DCorr metric produced a mean value of 26.8%, indicating that on average there was around 26.8% agreement between the diagnoses in the model-generated reports and the original diagnoses from the radiologist’s reports.

**Figure 6:**
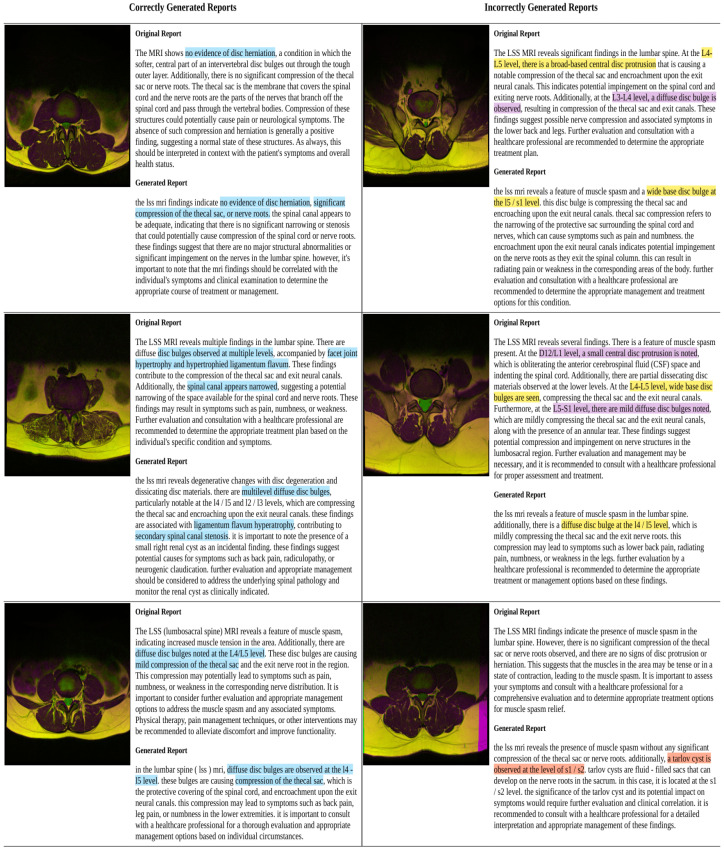
Comparative visualization of MRI reports from the GIT model and original versions. Blue highlights in the correctly generated reports section (**left**) indicate exact matches. In the incorrectly generated reports section (**right**), yellow represents mismatched terms, purple indicates missing terms in the original, and orange signifies missing terms in the Auto-Rad-generated report.

The empirical evaluation revealed that while the model is capable of generating coherent and grammatically structured reports, its performance in accurate diagnosis is relatively low. The DC and DCorr values indicate that there is room for improvement in capturing and aligning with the original diagnostic content. We believe that incorporating more diverse and varied data in the training process could potentially enhance the model’s diagnostic accuracy. Figure 6 and Table A1 illustrate some correctly and incorrectly generated reports using Auto-Rad.

### 4.3. Evaluation of Topic Distribution

Our topic-level evaluation on the test data reveals that the topic distribution of the generated reports is reasonably comparable to that of the original reports: JSD = 0.391 and EMD = 0.009. The JSD value suggests that while there are some differences between the topic distributions of the two sets, these differences are not substantial. The EMD value indicates a high similarity between the latent topic distributions of the original and generated reports, indicating that the generated reports capture the underlying topics in a manner very close to that of the original reports. These findings provide evidence that our model is able to generate key content that closely aligns with the topics present in the original reports.

## 5. Discussion

We have demonstrated that the GIT model effectively generates semantically accurate and coherent lumbar spine radiology reports, although some challenges remain in achieving high diagnostic precision. Despite these challenges, this section highlights the potential of deep learning-based ARRG systems in practical applications for lumbar spine diagnosis, especially if they reach robust performance levels. To comprehensively evaluate the GIT model’s capabilities, we compare it against other state-of-the-art ARRG models. Additionally, we discuss the limitations of our current approach and suggest strategies for future improvements. These enhancements aim to increase diagnostic accuracy and overall utility and advance the effectiveness of ARRG systems in lumbar spine diagnostics.

### 5.1. Comparison

In this section, we compare the performance of the GIT-base model with other ARRG models using NLG metrics. Metrics such as BLEU [36], ROUGE [35], and CIDEr [40] capture the quality of generated text in terms of semantic alignment and precision. The table below compares our GIT-base model with other ARRG system with different medical imaging modalities.

From the table, the GIT-base model performs best in the METEOR and ROUGE metrics, achieving scores of 0.3699 and 0.4570, respectively. Despite the GIT-base model’s strong performance in METEOR and ROUGE scores, it does not fully outperform other ARRG models, which is likely influenced by dataset size and the unique requirements of lumbar spine diagnostics. As shown in Table 2, our dataset includes only 1545 MRIs and 515 reports which is significantly fewer than the other ARRG systems. Another critical factor is the unique diagnostic demands of lumbar spine analysis. Unlike many ARRG models that focus on X-rays in a single plane, lumbar spine assessment typically requires both axial and sagittal views to capture comprehensive structural details and ensure diagnostic accuracy. In this study, we utilized only composite axial MRIs, which, while informative, provide limited perspectives for diagnosing lumbar conditions in full detail.

### 5.2. Limitations and Future Directions

A significant limitation of our study is the lack of comprehensive evaluation by radiologists or healthcare professionals, which limited our ability to conduct an expert assessment of the generated reports. While our empirical evaluations provide valuable insights, the accuracy and clinical relevance of novel diagnostics suggested by the model still require validation by radiologists with expertise in lumbar spine MRIs. Involving domain experts in verifying these generated reports would enhance the model’s utility and provide critical feedback for performance improvement. Incorporating frameworks such as reinforcement learning with human feedback [50] could be extended from LLMs to ARRG systems, allowing expert guidance to train more robust and clinically accurate models.

Additionally, the model faced challenges in accurately replicating specific diagnostic phrases from original reports, occasionally resulting in omissions of key details. These challenges likely stem from the limited sample size and the exclusion of sagittal MRI views, which are crucial for comprehensive lumbar spine assessment. Moreover, lumbar spine diagnostics often involve multimodal information, including electronic health records (EHRs) alongside imaging data. Developing a multimodal approach that integrates both comprehensive MRI views and EHR data would likely support a more robust and clinically informative system.

### 5.3. Utility of ARRG for Lumbar Spine Diagnosis

The primary goal of ARRG systems for lumbar spine diagnosis is to enhance, not replace, radiologists’ expertise by reducing the time and effort required for interpretation. Lumbar spine diagnostics are inherently time-intensive due to challenges like the lack of standardized criteria for conditions such as LSS [51], variability in imaging modalities, and the complex, dynamic nature of spinal disorders [52]. For instance, the absence of a clear consensus on LSS classification [51] often results in prolonged case interpretation and collaborative consultations. Accurate diagnosis frequently relies on multiple imaging modalities: T2-weighted MRI for canal and foraminal stenosis, CT for surgical planning, whole-spine X-rays for alignment, and roentgenkymography for assessing spinal instability [53]. Additionally, capturing both static (stenosis) and dynamic (instability) factors may require specialized imaging studies, such as myelography, to evaluate cerebrospinal fluid flow in weight-bearing positions [53].

An advanced ARRG system that integrates multimodal data, interpretability, and interactivity could significantly streamline these diagnostic processes. Although specific metrics for time and labor reduction in lumbar spine ARRG systems are not yet extensively documented, evidence suggests that DL-assisted diagnostics can greatly accelerate interpretation. For example, Lime et al. showed that DL-assisted radiologists reduced their interpretation time for LSS in spine MRIs from an average of 124–274 s to just 47–71 s [11]. Additionally, DL systems have demonstrated diagnostic accuracy comparable to that of radiologists, with Li et al. showing that DL models could diagnose LSS from CT scans with accuracy equivalent to specialists while also reducing assessment time [54].

However, existing DL systems often focus on individual conditions, such as LSS [11,54], disc herniation [55], or foraminal stenosis [12]. In contrast, a robust ARRG system has the potential to capture a wide range of lumbar spine conditions and present them in standardized, comprehensive reports. The advent of VL models, like GIT, opens new possibilities for extending image captioning and VQA tasks into ARRG and interactive dialogue systems for medical imaging.

## 6. Conclusions

We developed the Auto-Rad using GIT and large language models (LLMs) in assessing radiographs to assist radiologists. Our contributions are summarized below:

- We demonstrate that VL models, initially pre-trained using natural images for captioning, can be refined for assessing radiographs and generating reports for specific diseases (for example, LSS).
- We show that VL models can be fine-tuned with a small number of samples for generating diagnostic reports from radiographs.
- We built an end-to-end system (Auto-Rad) utilizing semistructured clinician notes, transforming them into coherent paragraphs for model training with the assistance of advanced language models.

In the future, we anticipate the participation of expert radiologists in the evaluation process of our model to provide a more comprehensive review. In addition, we plan to supplement our training set with more bias–variance balanced samples to improve Auto-Rad’s assessment capabilities. We also plan to implement expert in the loop, which will allow radiologist interaction with the model.

## Figures and Tables

**Table 1:** Comprehensive evaluation results of the Auto-Rad GIT-base model across three levels: (1) report generation quality assessed using NLG metrics; (2) diagnostic content accuracy measured through empirical metrics (DC, NDD, and DCorr); and (3) latent topic alignment evaluated with topic-level metrics.

Metric	Score	Type
ROUGE-1 (F1)	0.447	NLG
ROUGE-2 (F1)	0.185	NLG
ROUGE-L (F1)	0.299	NLG
ROUGE-Lsum (F1)	0.299	NLG
BLEU	0.110	NLG
METEOR	0.370	NLG
BERTScore (F1)	0.886	NLG
Perplexity	1.045	NLG
CIDEr	0.081	NLG
SPICE	0.288	NLG
DC	24.7%	Empirical
NDD	69.8%	Empirical
DCorr	26.8%	Empirical
JSD	0.391	Topic Level
EMD	0.009	Topic Level

**Table 2:** Performance comparison of automatic radiology report generation (ARRG) models with the GIT-base model. Most existing ARRG research has focused on X-rays or other imaging modalities, with few studies using MRI. The only ARRG work specific to lumbar spine MRI, as described by Han et al. [8], does not provide natural language generation (NLG) metrics and is therefore excluded from our comparison.

Model	Data (Images, Reports)	BLEU-1	BLEU-2	BLEU-3	BLEU-4	METEOR	ROUGE	CIDEr
GIT-base	Lumbar Spine MRI (1545, 515)	0.3827	0.1461	0.0676	0.0382	0.3699	0.4570	0.0805
CNN–LSTM–ATT [45]	Bladder Cancer (1000, 5000)	0.912	0.829	0.750	0.677	0.396	0.701	0.0204
CNN–HLSTM–DualLSTM–ATT [46]	IU X-Ray (7470, 3955)	0.373	0.246	0.175	0.126	0.163	0.315	0.359
CNN–HLSTM–RL [47]	MIMIC-CXR (327,281, 141,783)	0.313	0.206	0.146	0.103	0.146	0.306	1.046
Condition GPT2 [48]	IU X-Ray (7470, 7470)	0.387	0.245	0.166	0.111	0.164	0.289	0.257
Reinforce CNN–LSTM [49]	IU X-Ray (7470, 7470) and MIMIC-CXR	0.412	0.279	0.206	0.157	0.179	0.342	0.411

## Data Availability

The data used in this study are publicly available from the Mendeley Data repository at https://data.mendeley.com/datasets/k57fr854j2/2 (accessed on 1 September 2023). All data supporting the findings of this study can be accessed at this link.

## Appendix A. Examples of Generated Reports

In Table A1, we present a set of sample outputs categorized into two groups: correctly generated and incorrectly generated reports. The correctly generated reports are samples where the model successfully captured the key diagnostic elements, aligned with the original radiologist’s reports, and demonstrated clinically relevant terminology. In contrast, the incorrectly generated reports represent cases where the model failed to replicate critical diagnostic details or introduced inaccurate detail.

**Table A1:** Comparison of original and generated reports: the table presents examples of both accurate and inaccurate generated reports produced by the GIT-base model.

Original Report	Generated Report
**Correctly generated reports**	
The LSS (lumbosacral spine) MRI reveals the presence of a mild disc bulge but no significant stenosis.	The MRI of the lumbar spine shows no evidence of significant spinal stenosis or nerve root impingement.
The MRI findings reveal a feature of left-sided nerve root impingement with moderate compression due to a central disc protrusion.	The lumbar sacral spine MRI shows a right paracentral disc herniation with moderate nerve compression.
The lumbar sacral spine MRI reveals a specific feature of degenerative disc disease but no evidence of herniation.	The MRI scan reveals no evidence of disc herniation or nerve compression in the lumbar region.
Moderate disc bulge with mild impingement but no severe nerve compression is observed.	The MRI indicates a moderate disc bulge without severe nerve compression.
Mild bulging observed at L4-L5 level, not leading to severe spinal stenosis.	There is mild disc bulging noted at L4-L5 without significant stenosis.
Small disc protrusion at L3-L4 level but no nerve compression.	A small disc protrusion is noted at L3-L4, but it does not affect the nerve roots.
Degenerative changes seen in the lumbar spine but no signs of nerve impingement.	The lumbar spine shows degenerative changes without nerve root compression.
**Incorrectly generated reports**	
Evidence of severe canal stenosis at L3-L4 due to a large disc herniation.	The MRI scan is unremarkable with no significant findings.
Significant foraminal stenosis with nerve root compression detected on the left side.	No disc herniation or compression noted in the lumbar spine.
Central disc protrusion at L5-S1 with moderate thecal sac impingement.	The MRI shows normal alignment with no abnormalities.
Severe disc bulging at L4-L5 level with nerve compression noted.	Lumbar spine appears healthy with no signs of disc bulging.
The MRI reveals degenerative changes with significant disc herniation compressing the thecal sac.	No abnormalities detected in the MRI scan.
Diffuse disc bulge at multiple levels, leading to moderate stenosis.	No evidence of stenosis or disc bulging in the MRI.
Mild disc herniation at L2-L3 causing nerve root impingement.	MRI findings show no signs of nerve root compression or disc herniation.
